# 4-*tert*-Butyl-3′,4′-bis­(4-methyl­phen­yl)-3,4-dihydro-1*H*,4′*H*-spiro­[naphthalene-2,5′-[1,2]oxazol]-1-one

**DOI:** 10.1107/S1600536811025086

**Published:** 2011-06-30

**Authors:** Mohamed Akhazzane, Hafid Zouihri, A.Kella Bennani, Abdelali Kerbal, Ghali Al Houari

**Affiliations:** a Laboratoire de Chimie Organique, Faculté des Sciences Dhar el Mahraz, Université Sidi Mohammed Ben Abdellah, Fès, Morocco; b Laboratoire de Diffraction des Rayons X, Centre National pour la Recherche Scientifique et Technique, Rabat, Morocco

## Abstract

In the title compound, C_30_H_31_NO_2_, the cyclo­hexa­none ring in the naphthalene fused-ring system adopts a half-chair conformation, presumably due to conjugation of the benzene ring. The naphthalene ring system makes dihedral angles of 86.63 (7), 65.15 (8) and 63.18 (8)° with respect to the two methyl­benzene planes and the 1,2-oxazole ring system. Inter­molecular C—H⋯O and C—H⋯N hydrogen bonding and C—H⋯π inter­actions stabilize the crystal structure. The H atoms of the two methyl groups of the methyl­phenyl groups are disordered over two positions with equal occupancies.

## Related literature

For general background to dipolar-1,3 cyclo­addition reactions, see: Al Houari *et al.* (2008 ▶, 2010 ▶). For a related structure, see: Akhazzane *et al.* (2010 ▶). For puckering parameters, see: Cremer & Pople (1975 ▶).
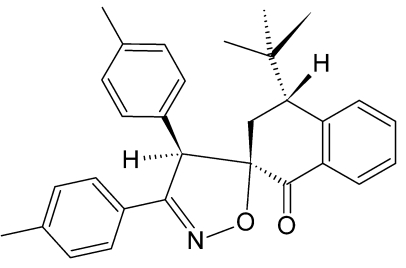


## Experimental

### 

#### Crystal data

C_30_H_31_NO_2_
*M* *_r_* = 437.56
Monoclinic, 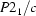

*a* = 6.9158 (2) Å
*b* = 25.0737 (5) Å
*c* = 13.8747 (3) Å
β = 94.359 (1)°
*V* = 2398.98 (10) Å^3^
*Z* = 4
Mo *K*α radiation
μ = 0.08 mm^−1^
*T* = 296 K
0.23 × 0.21 × 0.14 mm

#### Data collection

Bruker APEXII CCD detector diffractometer
25446 measured reflections
4473 independent reflections
3577 reflections with *I* > 2σ(*I*)
*R*_int_ = 0.028

#### Refinement

*R*[*F* ^2^ > 2σ(*F* ^2^)] = 0.040
*wR*(*F* ^2^) = 0.111
*S* = 1.06
4473 reflections
301 parameters
H-atom parameters constrained
Δρ_max_ = 0.16 e Å^−3^
Δρ_min_ = −0.15 e Å^−3^

### 

Data collection: *APEX2* (Bruker, 2005 ▶); cell refinement: *SAINT* (Bruker, 2005 ▶); data reduction: *SAINT*; program(s) used to solve structure: *SHELXS97* (Sheldrick, 2008 ▶); program(s) used to refine structure: *SHELXL97* (Sheldrick, 2008 ▶); molecular graphics: *PLATON* (Spek, 2009 ▶); software used to prepare material for publication: *publCIF* (Westrip, 2010 ▶).

## Supplementary Material

Crystal structure: contains datablock(s) I, global. DOI: 10.1107/S1600536811025086/fj2437sup1.cif

Structure factors: contains datablock(s) I. DOI: 10.1107/S1600536811025086/fj2437Isup2.hkl

Supplementary material file. DOI: 10.1107/S1600536811025086/fj2437Isup3.cml

Additional supplementary materials:  crystallographic information; 3D view; checkCIF report

## Figures and Tables

**Table 1:** Hydrogen-bond geometry (Å, °) *Cg*2 is the centroid of the C1–C6 ring.

*D*—H⋯*A*	*D*—H	H⋯*A*	*D*⋯*A*	*D*—H⋯*A*
C24—H24⋯O1^i^	0.93	2.54	3.4254 (17)	160
C29—H29*F*⋯N1^ii^	0.96	2.62	3.546 (2)	161
C26—H26*A*⋯*Cg*2^i^	0.96	2.77	3.6643 (16)	155

Symmetry codes: (i) 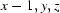; (ii) 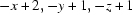.

**Fig. 1.**
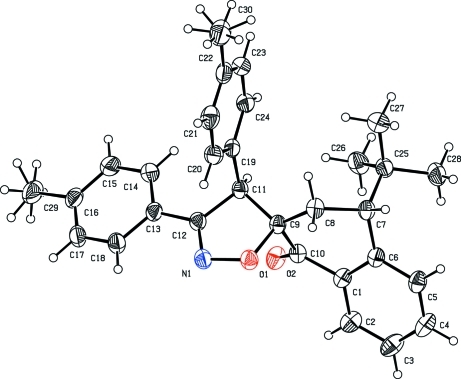
Molecular view of the title compound showing the atom-labeling scheme. Displacement ellipsoids are drawn at the 30% probability level. H atoms are represented as small spheres of arbitrary radii.

**Fig. 2.**
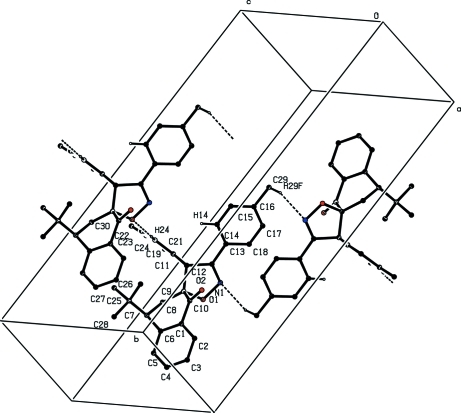
Partial packing view showing the chain formed by C—H···O and C—H···N hydrogen bondings. H atoms not involved in hydrogen bonds have been omitted for clarity.

## supplementary crystallographic information

### Comment

In this paper we studied the regiochemistry and stereochemistry in the reaction of the *p*-tolylnitriloxide with the 4-*tert*-butyl-2-(4-methylbenzylidene)-3,4-dihydronaphthalen-1-one.The X-ray crystal structure study shows that the carbonyl group is in position 5 of the isoxazoline. We also found out with this study, that the disposition of the ethyl group imposes an exclusive anti approach of the dipole. This stereochemistry is due to steric effects [Al Houari, *et al.* (2010) and Al Houari *et al.* (2008)].

In the title compound, as shown in Fig. 1, all bond lengths and angles are normal and comparable with those reported for the related structure [Akhazzane *et al.*, (2010)].

The cyclohexanone ring in the dihydronaphthalene fused-ring system adopts a half-chair conformation, presumably due to conjugation of the planar annulated benzo ring,, with the puckering parameters of: Q(2) = 0.5212 (14) Å, Phi(2) = 130.12 (15)°, Q(3) = 0.0845 (14) Å (Cremer & Pople, 1975). The dihedral angles between rings are 4'-methylbenzene (E) /naphthalene = 86.63 (7)°, 3'-methylbenzene (D) /naphthalene = 65.15 (8)°, oxazol (C) /naphthalene = 63.18 (8)°, 4'-methylbenzene (E) /3'-methylbenzene (D) = 75.77 (9)°, 3'-methylbenzene (D) /oxazol (C) = 14.55 (9)° and 4'-methylbenzene (E) /oxazol (C) = 89.68 (9)°.

The crystal packing of the title compound is illustrated in Fig. 2. Intermolecular C—H···O and C—H···N hydrogen bonding and C—H···π interactions stabilize the crystal structure.

### Experimental

In a100 ml flask, we dissolve 2 mmoles of the 4-*tert*-butyl-2-(4-methylbenzylidene)-3,4-dihydronaphthalen-1-one and 2.4 mmoles of *p*-tolyloxime in 20 ml of chloroform. The mixture is cooled to 0°C under magnetic stirring in an ice bath. Then 15 ml of bleach (NaOCl) at 18°Chl (chlorometric degree) is added in small doses without exceeding the temperature of 5°C. The mixture is left under magnetic stirring for 16 h at room temperature, then washed with water until pH is neutral and dried on sodium sulfate. The solvent is evaporated with a rotating evaporator and the oily residue is dissolved in ethanol. The precipitated cycloadduct is then recrystallized in ethanol.

### Refinement

The H atoms bound to C were treated as riding with their parent atoms [C—H distances are 0.93Å for CH groups and 0.97 Å for CH2 groups with *U*_iso_(H) = 1.2 *U*_eq_(C), and 0.96 Å for CH3 groups with *U*_iso_(H) = 1.5 *U*_eq_(C).

The H atoms of the two methyls of the methylbenzene systems are disordered over two positions with 0.5 equal occupancies.

### Crystal data

**Table:**

C_30_H_31_NO_2_	*F*(000) = 936
*M**_r_* = 437.56	*D*_x_ = 1.211 Mg m^−^^3^
Monoclinic, *P*2_1_/*c*	Mo *K*α radiation, λ = 0.71073 Å
Hall symbol: -P 2ybc	Cell parameters from 214 reflections
*a* = 6.9158 (2) Å	θ = 2.5–25.7°
*b* = 25.0737 (5) Å	µ = 0.08 mm^−^^1^
*c* = 13.8747 (3) Å	*T* = 296 K
β = 94.359 (1)°	Prism, colourless
*V* = 2398.98 (10) Å^3^	0.23 × 0.21 × 0.14 mm
*Z* = 4	

### Data collection

**Table:**

Bruker APEXII CCD detector diffractometer	3577 reflections with *I* > 2σ(*I*)
Radiation source: fine-focus sealed tube	*R*_int_ = 0.028
graphite	θ_max_ = 25.5°, θ_min_ = 2.2°
ω and φ scans	*h* = −8→8
25446 measured reflections	*k* = −30→28
4473 independent reflections	*l* = −16→16

### Refinement

**Table:**

Refinement on *F*^2^	Primary atom site location: structure-invariant direct methods
Least-squares matrix: full	Secondary atom site location: difference Fourier map
*R*[*F*^2^ > 2σ(*F*^2^)] = 0.040	Hydrogen site location: inferred from neighbouring sites
*wR*(*F*^2^) = 0.111	H-atom parameters constrained
*S* = 1.06	*w* = 1/[σ^2^(*F*_o_^2^) + (0.0553*P*)^2^ + 0.3646*P*] where *P* = (*F*_o_^2^ + 2*F*_c_^2^)/3
4473 reflections	(Δ/σ)_max_ = 0.001
301 parameters	Δρ_max_ = 0.16 e Å^−^^3^
0 restraints	Δρ_min_ = −0.15 e Å^−^^3^

### Special details

**Table:**

Geometry. All e.s.d.'s (except the e.s.d. in the dihedral angle between two l.s. planes) are estimated using the full covariance matrix. The cell e.s.d.'s are taken into account individually in the estimation of e.s.d.'s in distances, angles and torsion angles; correlations between e.s.d.'s in cell parameters are only used when they are defined by crystal symmetry. An approximate (isotropic) treatment of cell e.s.d.'s is used for estimating e.s.d.'s involving l.s. planes.
Refinement. Refinement of *F*^2^ against ALL reflections. The weighted *R*-factor *wR* and goodness of fit *S* are based on *F*^2^, conventional *R*-factors *R* are based on *F*, with *F* set to zero for negative *F*^2^. The threshold expression of *F*^2^ > σ(*F*^2^) is used only for calculating *R*-factors(gt) *etc*. and is not relevant to the choice of reflections for refinement. *R*-factors based on *F*^2^ are statistically about twice as large as those based on *F*, and *R*- factors based on ALL data will be even larger.

### Fractional atomic coordinates and isotropic or equivalent isotropic displacement parameters (Å^2^)

**Table:**

	*x*	*y*	*z*	*U*_iso_*/*U*_eq_	Occ. (<1)
C1	0.99029 (18)	0.74156 (5)	0.65946 (9)	0.0436 (3)	
C10	0.91627 (18)	0.68621 (5)	0.65774 (9)	0.0426 (3)	
C11	0.79579 (18)	0.60703 (5)	0.75229 (9)	0.0414 (3)	
C12	0.92787 (19)	0.56724 (5)	0.70820 (9)	0.0443 (3)	
C13	0.8637 (2)	0.51687 (5)	0.66211 (10)	0.0470 (3)	
C14	0.6692 (2)	0.50386 (6)	0.64952 (12)	0.0617 (4)	
C15	0.6080 (3)	0.45881 (7)	0.59815 (13)	0.0678 (5)	
C16	0.7374 (3)	0.42578 (6)	0.55695 (11)	0.0598 (4)	
C17	0.9322 (3)	0.43787 (6)	0.57194 (12)	0.0624 (4)	
C18	0.9957 (2)	0.48232 (6)	0.62346 (11)	0.0571 (4)	
C19	0.73938 (19)	0.58832 (5)	0.85013 (10)	0.0434 (3)	
C2	1.0839 (2)	0.76120 (6)	0.58097 (10)	0.0559 (4)	
C20	0.8776 (2)	0.56859 (6)	0.91866 (11)	0.0536 (4)	
C21	0.8290 (3)	0.55555 (6)	1.01065 (12)	0.0631 (4)	
C22	0.6423 (3)	0.56116 (6)	1.03785 (12)	0.0619 (4)	
C23	0.5045 (3)	0.57951 (6)	0.96882 (13)	0.0645 (4)	
C24	0.5510 (2)	0.59308 (6)	0.87645 (12)	0.0538 (4)	
C25	0.6435 (2)	0.76273 (6)	0.81758 (11)	0.0516 (4)	
C26	0.5354 (2)	0.73359 (7)	0.73308 (13)	0.0650 (4)	
C27	0.5627 (3)	0.74534 (8)	0.91206 (14)	0.0735 (5)	
C28	0.6084 (2)	0.82265 (6)	0.80518 (14)	0.0706 (5)	
C29	0.6694 (3)	0.37877 (7)	0.49570 (14)	0.0845 (6)	
C3	1.1560 (3)	0.81225 (7)	0.58255 (12)	0.0670 (5)	
C30	0.5917 (4)	0.54724 (8)	1.13904 (14)	0.0944 (7)	
C4	1.1346 (2)	0.84417 (6)	0.66174 (12)	0.0654 (4)	
C5	1.0423 (2)	0.82538 (6)	0.73955 (11)	0.0552 (4)	
C6	0.96796 (18)	0.77373 (5)	0.74004 (9)	0.0439 (3)	
C7	0.86674 (19)	0.75211 (5)	0.82404 (9)	0.0440 (3)	
C8	0.9315 (2)	0.69419 (5)	0.84267 (9)	0.0463 (3)	
C9	0.92760 (18)	0.65669 (5)	0.75533 (9)	0.0413 (3)	
H11	0.6796	0.6131	0.7088	0.050*	
H14	0.5781	0.5257	0.6759	0.074*	
H15	0.4765	0.4507	0.5914	0.081*	
H17	1.0227	0.4155	0.5466	0.075*	
H18	1.1278	0.4894	0.6325	0.069*	
H2	1.0974	0.7396	0.5274	0.067*	
H20	1.0046	0.5641	0.9025	0.064*	
H21	0.9247	0.5427	1.0553	0.076*	
H23	0.3768	0.5829	0.9846	0.077*	
H24	0.4546	0.6055	0.8317	0.065*	
H26A	0.3999	0.7423	0.7311	0.098*	
H26B	0.5518	0.6958	0.7414	0.098*	
H26C	0.5865	0.7444	0.6737	0.098*	
H27A	0.6311	0.7635	0.9652	0.110*	
H27B	0.5789	0.7075	0.9200	0.110*	
H27C	0.4274	0.7541	0.9104	0.110*	
H28A	0.6484	0.8338	0.7436	0.106*	
H28B	0.6820	0.8416	0.8557	0.106*	
H28C	0.4730	0.8301	0.8085	0.106*	
H29A	0.7795	0.3605	0.4734	0.127*	0.50
H29B	0.5876	0.3910	0.4412	0.127*	0.50
H29C	0.5977	0.3548	0.5335	0.127*	0.50
H29D	0.5303	0.3771	0.4920	0.127*	0.50
H29E	0.7223	0.3465	0.5242	0.127*	0.50
H29F	0.7122	0.3828	0.4319	0.127*	0.50
H3	1.2189	0.8252	0.5304	0.080*	
H30A	0.4561	0.5536	1.1447	0.142*	0.50
H30B	0.6667	0.5690	1.1850	0.142*	0.50
H30C	0.6201	0.5103	1.1517	0.142*	0.50
H30D	0.7058	0.5350	1.1762	0.142*	0.50
H30E	0.4952	0.5196	1.1359	0.142*	0.50
H30F	0.5418	0.5783	1.1692	0.142*	0.50
H4	1.1829	0.8788	0.6628	0.078*	
H5	1.0295	0.8475	0.7925	0.066*	
H7	0.9198	0.7722	0.8805	0.053*	
H8A	1.0628	0.6948	0.8726	0.056*	
H8B	0.8496	0.6789	0.8892	0.056*	
N1	1.10645 (17)	0.58119 (5)	0.71418 (9)	0.0514 (3)	
O1	1.12294 (13)	0.63264 (4)	0.75739 (7)	0.0499 (2)	
O2	0.86056 (16)	0.66355 (4)	0.58328 (7)	0.0569 (3)	

### Atomic displacement parameters (Å^2^)

**Table:**

	*U*^11^	*U*^22^	*U*^33^	*U*^12^	*U*^13^	*U*^23^
C1	0.0448 (7)	0.0429 (7)	0.0412 (7)	−0.0023 (6)	−0.0079 (5)	0.0048 (6)
C10	0.0445 (7)	0.0415 (7)	0.0409 (7)	0.0014 (5)	−0.0029 (5)	−0.0014 (6)
C11	0.0422 (7)	0.0366 (7)	0.0445 (7)	0.0035 (5)	−0.0023 (5)	0.0010 (5)
C12	0.0498 (8)	0.0392 (7)	0.0440 (7)	0.0043 (6)	0.0044 (6)	0.0039 (6)
C13	0.0606 (8)	0.0366 (7)	0.0446 (7)	0.0026 (6)	0.0088 (6)	0.0048 (6)
C14	0.0647 (10)	0.0493 (9)	0.0736 (10)	−0.0051 (7)	0.0216 (8)	−0.0118 (8)
C15	0.0722 (10)	0.0571 (10)	0.0766 (11)	−0.0160 (8)	0.0223 (9)	−0.0129 (8)
C16	0.0908 (12)	0.0401 (8)	0.0512 (8)	−0.0094 (8)	0.0237 (8)	0.0015 (7)
C17	0.0853 (12)	0.0412 (9)	0.0632 (10)	0.0099 (8)	0.0217 (8)	−0.0014 (7)
C18	0.0638 (9)	0.0456 (9)	0.0625 (9)	0.0088 (7)	0.0089 (7)	0.0012 (7)
C19	0.0464 (7)	0.0337 (7)	0.0503 (7)	0.0020 (5)	0.0039 (6)	−0.0003 (6)
C2	0.0660 (9)	0.0596 (10)	0.0406 (7)	−0.0096 (7)	−0.0053 (6)	0.0056 (6)
C20	0.0540 (8)	0.0505 (9)	0.0563 (9)	0.0076 (6)	0.0050 (7)	0.0095 (7)
C21	0.0843 (11)	0.0491 (9)	0.0550 (9)	0.0057 (8)	−0.0013 (8)	0.0083 (7)
C22	0.0949 (12)	0.0360 (8)	0.0576 (9)	−0.0022 (8)	0.0232 (9)	−0.0027 (7)
C23	0.0694 (10)	0.0493 (9)	0.0787 (11)	−0.0011 (7)	0.0312 (9)	−0.0031 (8)
C24	0.0487 (8)	0.0451 (8)	0.0678 (10)	0.0022 (6)	0.0064 (7)	0.0001 (7)
C25	0.0489 (8)	0.0453 (8)	0.0607 (9)	0.0037 (6)	0.0048 (6)	−0.0041 (7)
C26	0.0449 (8)	0.0682 (11)	0.0803 (11)	0.0065 (7)	−0.0069 (7)	−0.0111 (9)
C27	0.0732 (11)	0.0704 (12)	0.0799 (12)	0.0003 (9)	0.0245 (9)	−0.0038 (9)
C28	0.0612 (9)	0.0518 (10)	0.0990 (13)	0.0134 (7)	0.0066 (9)	−0.0023 (9)
C29	0.1239 (17)	0.0581 (11)	0.0761 (12)	−0.0256 (11)	0.0377 (11)	−0.0179 (9)
C3	0.0802 (11)	0.0661 (11)	0.0533 (9)	−0.0224 (9)	−0.0051 (8)	0.0146 (8)
C30	0.161 (2)	0.0620 (12)	0.0659 (11)	−0.0026 (12)	0.0459 (13)	−0.0033 (9)
C4	0.0730 (10)	0.0488 (9)	0.0720 (11)	−0.0180 (8)	−0.0103 (8)	0.0118 (8)
C5	0.0579 (8)	0.0439 (8)	0.0620 (9)	−0.0050 (6)	−0.0070 (7)	−0.0035 (7)
C6	0.0409 (7)	0.0402 (7)	0.0488 (7)	0.0004 (5)	−0.0084 (6)	0.0016 (6)
C7	0.0492 (7)	0.0398 (7)	0.0419 (7)	−0.0003 (6)	−0.0045 (6)	−0.0047 (6)
C8	0.0527 (7)	0.0443 (8)	0.0402 (7)	0.0017 (6)	−0.0070 (6)	0.0004 (6)
C9	0.0415 (7)	0.0379 (7)	0.0432 (7)	0.0042 (5)	−0.0044 (5)	0.0020 (5)
N1	0.0517 (7)	0.0434 (7)	0.0590 (7)	0.0060 (5)	0.0046 (5)	−0.0015 (5)
O1	0.0421 (5)	0.0447 (6)	0.0620 (6)	0.0031 (4)	−0.0028 (4)	−0.0024 (4)
O2	0.0773 (7)	0.0498 (6)	0.0420 (5)	−0.0073 (5)	−0.0060 (5)	−0.0044 (4)

### Geometric parameters (Å, °)

**Table:**

C1—C10	1.4788 (18)	C26—H26A	0.9600
C1—C2	1.398 (2)	C27—H27C	0.9600
C1—C6	1.3969 (19)	C27—H27B	0.9600
C10—C9	1.5399 (18)	C27—H27A	0.9600
C11—H11	0.9800	C28—H28C	0.9600
C11—C19	1.5150 (18)	C28—H28B	0.9600
C11—C12	1.5129 (18)	C28—H28A	0.9600
C13—C12	1.4689 (19)	C29—H29F	0.9600
C13—C18	1.395 (2)	C29—H29E	0.9600
C13—C14	1.383 (2)	C29—H29D	0.9600
C14—H14	0.9300	C29—H29C	0.9600
C14—C15	1.385 (2)	C29—H29B	0.9600
C15—H15	0.9300	C29—H29A	0.9600
C16—C29	1.507 (2)	C3—H3	0.9300
C16—C17	1.381 (2)	C3—C4	1.377 (2)
C16—C15	1.375 (2)	C30—H30F	0.9600
C17—H17	0.9300	C30—H30E	0.9600
C18—H18	0.9300	C30—H30D	0.9600
C18—C17	1.378 (2)	C30—H30C	0.9600
C19—C20	1.3871 (19)	C30—H30B	0.9600
C19—C24	1.3845 (19)	C30—H30A	0.9600
C2—H2	0.9300	C4—H4	0.9300
C2—C3	1.373 (2)	C5—H5	0.9300
C20—H20	0.9300	C5—C4	1.378 (2)
C20—C21	1.383 (2)	C6—C5	1.394 (2)
C21—H21	0.9300	C7—H7	0.9800
C22—C30	1.513 (2)	C7—C25	1.5625 (19)
C22—C21	1.380 (3)	C7—C8	1.5357 (18)
C22—C23	1.378 (3)	C7—C6	1.5055 (19)
C23—H23	0.9300	C8—H8B	0.9700
C24—H24	0.9300	C8—H8A	0.9700
C24—C23	1.387 (2)	C9—C11	1.5420 (18)
C25—C28	1.530 (2)	C9—C8	1.5324 (18)
C25—C26	1.528 (2)	N1—O1	1.4236 (15)
C25—C27	1.527 (2)	N1—C12	1.2802 (18)
C26—H26C	0.9600	O1—C9	1.4777 (15)
C26—H26B	0.9600	O2—C10	1.2156 (15)
			
C2—C1—C10	119.88 (12)	C25—C28—H28B	109.5
C6—C1—C10	119.72 (12)	C25—C28—H28A	109.5
C6—C1—C2	120.39 (13)	H29E—C29—H29F	109.5
C1—C10—C9	116.26 (11)	H29D—C29—H29F	109.5
O2—C10—C9	120.96 (12)	H29C—C29—H29F	141.1
O2—C10—C1	122.63 (12)	H29B—C29—H29F	56.3
C9—C11—H11	110.2	H29A—C29—H29F	56.3
C19—C11—H11	110.2	C16—C29—H29F	109.5
C12—C11—H11	110.2	H29D—C29—H29E	109.5
C19—C11—C9	114.68 (10)	H29C—C29—H29E	56.3
C12—C11—C9	99.77 (10)	H29B—C29—H29E	141.1
C12—C11—C19	111.26 (10)	H29A—C29—H29E	56.3
C13—C12—C11	124.84 (12)	C16—C29—H29E	109.5
N1—C12—C11	113.75 (12)	H29C—C29—H29D	56.3
N1—C12—C13	121.40 (12)	H29B—C29—H29D	56.3
C18—C13—C12	121.11 (13)	H29A—C29—H29D	141.1
C14—C13—C12	121.10 (13)	C16—C29—H29D	109.5
C14—C13—C18	117.64 (14)	H29B—C29—H29C	109.5
C15—C14—H14	119.6	H29A—C29—H29C	109.5
C13—C14—H14	119.6	C16—C29—H29C	109.5
C13—C14—C15	120.90 (15)	H29A—C29—H29B	109.5
C14—C15—H15	119.2	C16—C29—H29B	109.5
C16—C15—H15	119.2	C16—C29—H29A	109.5
C16—C15—C14	121.51 (16)	C4—C3—H3	120.2
C17—C16—C29	121.05 (16)	C2—C3—H3	120.2
C15—C16—C29	121.35 (17)	C2—C3—C4	119.58 (15)
C15—C16—C17	117.58 (15)	H30E—C30—H30F	109.5
C16—C17—H17	119.2	H30D—C30—H30F	109.5
C18—C17—H17	119.2	H30C—C30—H30F	141.1
C18—C17—C16	121.64 (15)	H30B—C30—H30F	56.3
C13—C18—H18	119.7	H30A—C30—H30F	56.3
C17—C18—H18	119.7	C22—C30—H30F	109.5
C17—C18—C13	120.66 (15)	H30D—C30—H30E	109.5
C20—C19—C11	120.91 (12)	H30C—C30—H30E	56.3
C24—C19—C11	121.41 (12)	H30B—C30—H30E	141.1
C24—C19—C20	117.58 (13)	H30A—C30—H30E	56.3
C1—C2—H2	119.8	C22—C30—H30E	109.5
C3—C2—H2	119.8	H30C—C30—H30D	56.3
C3—C2—C1	120.33 (15)	H30B—C30—H30D	56.3
C19—C20—H20	119.5	H30A—C30—H30D	141.1
C21—C20—H20	119.5	C22—C30—H30D	109.5
C21—C20—C19	120.91 (14)	H30B—C30—H30C	109.5
C20—C21—H21	119.1	H30A—C30—H30C	109.5
C22—C21—H21	119.1	C22—C30—H30C	109.5
C22—C21—C20	121.75 (15)	H30A—C30—H30B	109.5
C21—C22—C30	121.17 (18)	C22—C30—H30B	109.5
C23—C22—C30	121.71 (18)	C22—C30—H30A	109.5
C23—C22—C21	117.12 (15)	C5—C4—H4	119.6
C24—C23—H23	119.1	C3—C4—H4	119.6
C22—C23—H23	119.1	C3—C4—C5	120.72 (15)
C22—C23—C24	121.85 (15)	C6—C5—H5	119.5
C23—C24—H24	119.6	C4—C5—H5	119.5
C19—C24—H24	119.6	C4—C5—C6	120.97 (14)
C19—C24—C23	120.75 (15)	C1—C6—C7	119.85 (12)
C28—C25—C7	108.75 (12)	C5—C6—C7	122.14 (13)
C26—C25—C7	112.72 (11)	C5—C6—C1	118.00 (13)
C27—C25—C7	109.05 (13)	C25—C7—H7	105.4
C26—C25—C28	108.63 (13)	C8—C7—H7	105.4
C27—C25—C28	108.17 (13)	C6—C7—H7	105.4
C27—C25—C26	109.42 (13)	C8—C7—C25	116.43 (11)
H26B—C26—H26C	109.5	C6—C7—C25	114.19 (11)
H26A—C26—H26C	109.5	C6—C7—C8	108.91 (11)
C25—C26—H26C	109.5	H8A—C8—H8B	107.2
H26A—C26—H26B	109.5	C7—C8—H8B	108.0
C25—C26—H26B	109.5	C9—C8—H8B	108.0
C25—C26—H26A	109.5	C7—C8—H8A	108.0
H27B—C27—H27C	109.5	C9—C8—H8A	108.0
H27A—C27—H27C	109.5	C9—C8—C7	117.37 (10)
C25—C27—H27C	109.5	C10—C9—C11	111.94 (10)
H27A—C27—H27B	109.5	C8—C9—C11	119.46 (11)
C25—C27—H27B	109.5	O1—C9—C11	102.04 (10)
C25—C27—H27A	109.5	C8—C9—C10	113.40 (11)
H28B—C28—H28C	109.5	O1—C9—C10	101.45 (10)
H28A—C28—H28C	109.5	O1—C9—C8	105.91 (9)
C25—C28—H28C	109.5	C12—N1—O1	108.71 (11)
H28A—C28—H28B	109.5	N1—O1—C9	108.53 (9)

### Hydrogen-bond geometry (Å, °)

**Table:**

Cg2 is the centroid of the C1–C6 ring.

**Table:**

*D*—H···*A*	*D*—H	H···*A*	*D*···*A*	*D*—H···*A*
C24—H24···O1^i^	0.93	2.54	3.4254 (17)	160
C29—H29F···N1^ii^	0.96	2.62	3.546 (2)	161
C26—H26A···Cg2^i^	0.96	2.77	3.6643 (16)	155

Symmetry codes: (i) *x*−1, *y*, *z*; (ii) −*x*+2, −*y*+1, −*z*+1.

## References

[bb1] Akhazzane, M., Zouihri, H., Daran, J.-C., Kerbal, A. & Al Houari, G. (2010). *Acta Cryst.* E**66**, o3067. 10.1107/S1600536810044387 PMC3011461 21589377

[bb2] Al Houari, G., Baba, M. F., Miqueu, K., Sotiropoulos, J. M., Garrigues, B., Benhadda, T., Benlarbi, N., Safir, I. & et Kerbal, A. (2008). *J. Mar. Chim. Heterocycl.* **7**, 16–20.

[bb3] Al Houari, G., Bennani, A. K., Bennani, B., Daoudi, M., Benlarbi, N., El Yazidi, M., Garrigues, B. & Kerbal, A. (2010). *J. Mar. Chim. Heterocycl.* **9**, 36–43.

[bb4] Bruker (2005). *APEX2* and *SAINT* Bruker AXS Inc., Madison, Wisconsin, USA.

[bb5] Cremer, D. & Pople, J. A. (1975). *J. Am. Chem. Soc.* **97**, 1354-1358;

[bb6] Sheldrick, G. M. (2008). *Acta Cryst.* A**64**, 112–122. 10.1107/S0108767307043930 18156677

[bb7] Spek, A. L. (2009). *Acta Cryst.* D**65**, 148–155. 10.1107/S090744490804362X PMC2631630 19171970

[bb8] Westrip, S. P. (2010). *J. Appl. Cryst.* **43**, 920–925.

